# Sub-Chronic Ketamine Administration Increases Dopamine Synthesis Capacity in the Mouse Midbrain: a Preclinical *In Vivo* PET Study

**DOI:** 10.1007/s11307-023-01865-y

**Published:** 2023-10-23

**Authors:** Alice Petty, Anna Garcia-Hidalgo, Els F Halff, Sridhar Natesan, Dominic J Withers, Elaine E Irvine, Michelle Kokkinou, Lisa A Wells, David R Bonsall, Sac-Pham Tang, Mattia Veronese, Oliver D Howes

**Affiliations:** 1 Faculty of Medicine, Imperial College London, Institute of Clinical Sciences, London, UK; 2 Psychiatric Imaging Group, MRC London Institute of Medical Sciences, London, UK; 3 King’s College London, Institute of Psychiatry, Psychology and Neuroscience, London, UK; 4 Metabolic Signalling Group, MRC London Institute of Medical Sciences, London, UK; 5 Invicro, Burlington Danes, Hammersmith Hospital, London, UK; 6 Department of Neuroimaging, Institute of Psychiatry, Psychology and Neuroscience, King’s College London, London, UK; 7 Department of Information Engineering, University of Padua, Padua, Italy; 8 South London and Maudsley NHS Foundation Trust, Camberwell, London, UK; 9 H. Lundbeck A/S, St Albans, AL1 2PS UK

**Keywords:** Dopamine, Preclinical, PET, Midbrain, Schizophrenia, Ketamine, F-DOPA

## Abstract

**Purpose:**

There is robust evidence that people with schizophrenia show elevated dopamine (DA) synthesis capacity in the striatum. This finding comes from positron emission tomography (PET) studies using radiolabelled l-3,4-dihydroxyphenylalanine (^18^F-DOPA). DA synthesis capacity also appears to be elevated in the midbrain of people with schizophrenia compared to healthy controls. We therefore aimed to optimise a method to quantify ^18^F-DOPA uptake in the midbrain of mice, and to utilise this method to quantify DA synthesis capacity in the midbrain of the sub-chronic ketamine model of schizophrenia-relevant hyperdopaminergia.

**Procedures:**

Adult male C57Bl6 mice were treated daily with either ketamine (30 mg/kg, i.p.) or vehicle (saline) for 5 days. On day 7, animals were administered ^18^F-DOPA (i.p.) and scanned in an Inveon PET/CT scanner. Data from the saline-treated group were used to optimise an atlas-based template to position the midbrain region of interest and to determine the analysis parameters which resulted in the greatest intra-group consistency. These parameters were then used to compare midbrain DA synthesis capacity (*K*_i_^Mod^) between ketamine- and saline-treated animals.

**Results:**

Using an atlas-based template to position the 3.7 mm^3^ midbrain ROI with a T*–*T*^end^ window of 15–140 min to estimate *K*_i_^Mod^ resulted in the lowest intra-group variability and moderate test–retest agreement. Using these parameters, we found that *K*_i_^Mod^ was elevated in the midbrain of ketamine-treated animals in comparison to saline-treated animals (*t*_(22)_ = 2.19, *p* = 0.048). A positive correlation between DA synthesis capacity in the striatum and the midbrain was also evident in the saline-treated animals (*r*^2^ = 0.59, *p* = 0.005) but was absent in ketamine-treated animals (*r*^2^ = 0.004, *p* = 0.83).

**Conclusions:**

Using this optimised method for quantifying ^18^F-DOPA uptake in the midbrain, we found that elevated striatal DA synthesis capacity in the sub-chronic ketamine model extends to the midbrain. Interestingly, the dysconnectivity between the midbrain and striatum seen in this model is also evident in the clinical population. This model may therefore be ideal for assessing novel compounds which are designed to modulate pre-synaptic DA synthesis capacity.

**Supplementary Information:**

The online version contains supplementary material available at 10.1007/s11307-023-01865-y.

## Introduction

Ketamine is an antagonist of the glutamatergic N-methyl-d-aspartate (NMDA) receptor. NMDA receptor hypofunction may contribute to the neurobiology of schizophrenia [1, 2], and ketamine can be used to generate a preclinical model with relevance to schizophrenia [3–6]. When given sub-chronically at sub-anaesthetic doses, rodents display behavioural and neurochemical phenotypes relevant to schizophrenia [3, 7], and we have previously shown that the sub-chronic ketamine model results in elevated [^18^F]-l-3,4-dihydroxyphenylalanine (^18^F-DOPA) uptake in the striatum in mice [6, 8]. This mimics the increase in ^18^F-DOPA uptake in the striatum which is evident in people with schizophrenia [9–11]. This finding likely reflects enhanced dopamine (DA) synthesis capacity in the striatum and demonstrates that the sub-chronic ketamine model replicates this key aspect of schizophrenia neurobiology.

Dopaminergic innervation to the striatum comes from neurons located in the midbrain, specifically in the substantia nigra pars compacta (SNpc) and the ventral tegmental area (VTA; [12]). Dopaminergic dysfunction in the striatum may therefore originate in the midbrain. Indeed, there is now neuroimaging and post-mortem evidence of dopaminergic dysfunction in the midbrain of people with schizophrenia [13–15]. For instance, a recent meta-analysis found an increase in neuromelanin in the midbrain of people with schizophrenia compared to healthy controls [14]. Neuromelanin is a product of excess cytosolic DA and its precursor 3,4-dihydroxyphenylalanine (DOPA) [16], and this is therefore a strong indication that DA synthesis in the midbrain is elevated in people with schizophrenia. Additionally, an ^18^F-DOPA PET study found that DA synthesis capacity was elevated in the nigra of people with schizophrenia compared to healthy controls, as well as in the striatum [13].

In the preclinical ketamine model, striatal DA dysfunction (and the hyperlocomotor phenotype) was abolished when midbrain DA neuron activity was inhibited chemogenetically [6]. This finding suggests that midbrain DA neuron function contributes to the neurochemical and behavioural phenotypes of this model. However, it is unknown whether ketamine directly affects DA synthesis capacity in the midbrain, or whether it acts via indirect mechanisms. Understanding this relationship between dopaminergic regions in this model with relevance to schizophrenia has implications for understanding the dopaminergic dysfunction in this disorder.

Within the rodent brain, the striata show the strongest ^18^F-DOPA signal, and the experimental parameters for estimation of DA synthesis capacity in this region are published elsewhere [6, 8, 17]. However, the considerable differences in size and physiology between the striatum and the midbrain—including an elevated ratio of DA to its metabolite 3,4-dihydroxyphenylacetic acid (DOPAC) in the midbrain compared to the striatum [18]—mean that we cannot presume to use the same analytical parameters for ^18^F-DOPA quantification in both compartments. This work therefore has two aims. The first is to establish a method to place the midbrain region of interest accurately based on the acquired CT structural scan and to define the optimal analysis parameters which allow for robust, reliable quantification of ^18^F-DOPA signal in the mouse midbrain. The second aim of this work is to use this method to determine whether the sub-chronic ketamine model displays elevated DA synthesis capacity in the midbrain alongside the striatum.

## Methods

### Subjects

The analysis of ^18^F-DOPA uptake in the striatum has already been published for this dataset (for both the saline- and ketamine-treated animals and the test–retest cohort) [8]. These scans were re-analysed here to assess ^18^F-DOPA uptake in the midbrain, and therefore no new animals were used for this study. The details below refer to the original cohorts of animals. Wild-type male C57BL/6J mice were acquired from Charles River (Kent, UK). Animals were housed in groups of up to 4 and provided with *ad libitum* food and water. Animals were acclimatised to the facility for at least 1 week prior to any experimental procedures, which were undertaken in adult animals (8–12 weeks old). For animal weights, see Table 1. All animal experimental procedures were performed in accordance with the UK Animals (Scientific Procedures) Act 1986 and EU directive 2010/63/EU, and protocols were approved by the Imperial College Animal Welfare and Ethical Review Body and conducted under the licence PE0206466.

**Table 1:** Animal weights and injected ^18^F-DOPA dose for the saline and ketamine-treated cohorts, and the test–retest cohort. Mean (SD)

	Saline	Ketamine	*T*-test
*n*	12	12	-
Weight (g)	25.88 (1.3)	25.11 (2.0)	*t*_(23)_ = 1.18, *p* = 0.27
Dose injected (MBq)	12.44 (4.8)	9.64 (4.6)	*t*_(23)_ =1.46, *p* = 0.15
	Test	Retest	*T*-test
*n*	6		*-*
Weight (g)	24.22 (0.7)	23.28 (1.2)	*t*_(5)_ =2.4, *p* = 0.06
Dose injected (MBq)	15.72 (3.1)	14.28 (7.5)	*t*_(5)_ =0.63, *p* = 0.55

*MBq* megabecquerel

### Experimental Cohorts

#### Saline-Treated Cohort

Animals were administered saline intraperitoneally (i.p.; 0.9%) once daily for 5 consecutive days, as they formed the control cohort for the ketamine treatment (see below). Animals then underwent the PET scan on day 7. Animals were euthanized immediately after the scan. Although 14 animals were originally treated with saline, 2 animals were excluded from analysis due to issues with the scan data, resulting in a final *n* = 12 for this group. This cohort was used to establish the midbrain template and the analysis parameters which resulted in the best-fitting data and lowest intra-group variability when estimating ^18^F-DOPA uptake in the midbrain.

#### Ketamine-Treated Cohort

To generate the sub-chronic ketamine model, ketamine hydrochloride (Sigma-Aldrich, K2753) was dissolved in 0.9% saline solution and administered to mice (*n* = 12) at a dose of 30 mg/kg (i.p) once daily for 5 consecutive days. On day 7, after 2 days of washout, animals underwent an ^18^F-DOPA preclinical PET scan. Animals were euthanized immediately after the scan.

#### Test-Retest Cohort

A separate cohort of untreated mice (*n* = 6) was scanned twice to assess reproducibility of results. Animals underwent the initial scan (“test”) and were then recovered from the anaesthesia. They were re-scanned (“retest”) approximately 48 h later, after which they were immediately euthanized.

### PET Acquisition

^18^F-DOPA was synthesised as described previously [19]. One hour prior to the administration of ^18^F-DOPA, mice were anaesthetised with isoflurane (4%), which was maintained at 1–2% for the duration of the scan (O_2_ 1 L/min). Respiration rate and body temperature were continuously monitored (BioVet, m2m Imaging Corp, OH, USA). To prevent peripheral metabolism of ^18^F-DOPA, inhibitors of catechol-O-methyl-transferase (COMT) and aromatic amino acid decarboxylase (AADC)—entacapone (40 mg/kg; Sigma-Aldrich, SML0654) and benserazide hydrochloride (10 mg/kg; Sigma-Aldrich, B7283)—were given i.p. 45 min and 30 min prior to the administration of ^18^F-DOPA, respectively [6, 17, 20]. To avoid competition with the uptake of other neutral amino acids in the diet, animals were fasted for 45 min before being anaesthetised [6, 21]. An i.p. cannula used to deliver the radiotracer was inserted 30 min prior to the scan. The mice were then placed into an Inveon PET/CT scanner (Siemens, Surrey, UK) and underwent a 10-min CT scan to allow for attenuation correction of the PET signal. A dynamic PET scan was then started concomitantly with the delivery of ^18^F-DOPA via the i.p. cannula, and data were collected for 140 min. The test–retest datasets were only acquired up to 120 min, due to changes in the scanning parameters between cohorts. The average ^18^F-DOPA doses delivered for each experimental cohort are provided in Table 1.

### PET Image Processing

Following acquisition, data were histogrammed into 45 frames (10 × 3 s, 6 × 5 s, 8 × 30 s, 5 × 60 s, 6 × 300 s, 8 × 600s for data acquired up to 120 min). For data acquired up to 140 min, an additional 2 × 600 s frames were added. Data were reconstructed using filtered back projection with CT attenuation correction, adjusting for random noise, scatter, and radiotracer decay. Image processing was carried out using the Inveon Research Workspace software (Siemens, USA). The CT image was manually co-registered to the reconstructed PET image using the outline of the skull as a reference. For each subject, the percentage-injected dose was normalised for body weight and injected activity to provide standardised uptake values (SUV). The striatal (0.70 mm^3^) and cerebellar (the reference region; rectangular; 1.1 mm^3^) regions of interest (ROI) were placed manually on summation radioactivity images guided by CT and mouse brain anatomy based on previous studies [6, 8, 17].

#### Developing the Midbrain Template

**Fig. 1:**
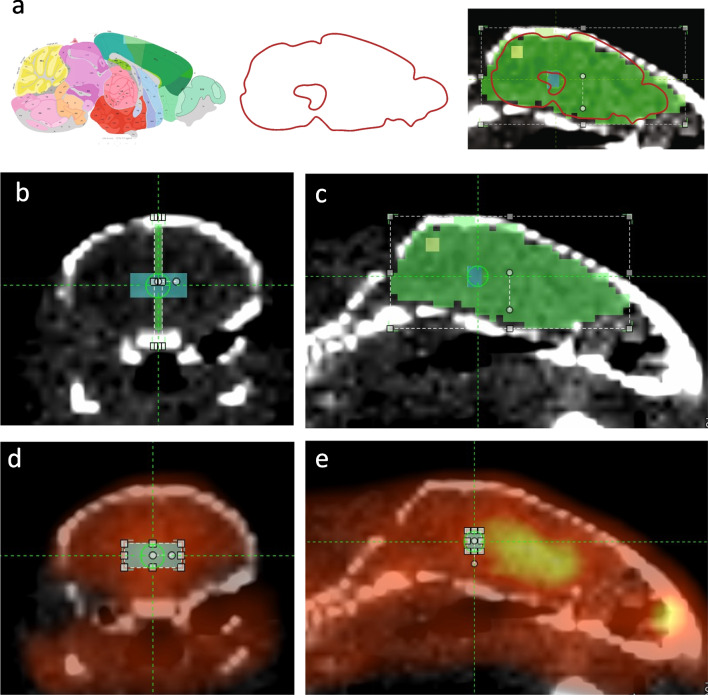
Developing the midbrain template. **a** An atlas image of the mousebrain sagittal midline (from the Allen Mouse Brain Atlas) was used to identify the midbrain region, and a mask based on this atlas image (green) was aligned to an ROI drawn within the outline of the skull from a representative mouse CT scan. The cerebellum (yellow) was placed in the posterior part of the brain. This template was then used to place the 3.7-mm^3^ midbrain ROI (blue), seen from the **b** coronal and **c** sagittal perspective. The ^18^F-DOPA signal (yellow-red spectrum) can be seen in the **d** coronal and **e** sagittal planes, along with the placement of the midbrain ROI

Based on stereotaxic coordinates from the Paxinos Mouse Brain Atlas, the volume of the midbrain region which incorporates the bilateral SNpc and VTA is approximately 3.2 mm^3^ [22] (Supp. Figure S1a). Therefore, we established a rectangular midbrain ROI of 3.7 mm^3^ (16 voxels, Supp. Table S1). A template was developed to standardise the placement of this ROI in the brain; a representative CT image at the midline sagittal viewpoint was aligned with a mask based on the sagittal P56 Allen Mouse Brain Atlas, image 21 (mouse.brain-map.org/static/atlas [23]) to identify the location of the midbrain (Fig. 1). The resulting template was applied to the CT images of each animal to place the midbrain ROI. Supplementary figure S1b shows the placements for all 12 saline-treated animals.

### Modified Patlak Analysis

The kinetic analysis to provide an estimate of the rate of ^18^F-DOPA uptake was performed using a MATLAB pipeline developed in-house (version R2022a; MathWorks, MA, USA). A modified Patlak graphical analysis was used to determine the influx rate constant *K*_i_^Mod^ (min^−1^). *K*_i_^Mod^ is a modified version of the influx rate constant *K*_i_^Std^ (min^−1^) and takes into account an estimation of *k*_loss_ (min^−1^), the rate at which signal is removed from the system due to metabolism of ^18^F-dopamine, as described previously [20, 24–26]. To establish the optimal time window for estimating *K*_i_^Mod^ in the midbrain, data from the saline-treated animals were processed through the modified Patlak analysis pipeline using varying start (T*) and end times (*T*^end^). The analysis pipeline also quantifies the coefficient of variation (CV%) for the Patlak modelling, which gives an indication of how well the fitting has worked. These numbers were also compared for each T*–*T*^end^ window, with the lowest CV% indicating the best fit of the data to the graphical model. In some instances, the Patlak model can fail due to excessive noise in the data, or too few usable data points. This requires these datasets to be excluded, and therefore the number of animals included for each T*–*T*^end^ window (as a fraction of the whole group) is also noted.

### Statistical Analysis

Statistical tests were carried out using GraphPad Prism v9 (GraphPad Software, La Jolla, California, USA). Data were tested for statistical outliers (Grubbs, *α* = 0.05) and normality (Kolmogorov-Smirnov test). Significance was defined as *α* < 0.05. Data is always presented as mean ± standard deviation (SD). SD of the saline-treated cohort was compared for each T*–*T*^end^ time windows to determine the parameters which produced the least within-group variability. *T*-test was used to compare mean differences between groups, with Welch’s correction used where variance was significantly different between groups. Consistency between the test–retest scans was analysed using an intra-class correlation coefficient (ICC; using a two-way mixed model with absolute agreement) and absolute percentage variance (%). Pearson’s correlations coefficients were calculated to assess significant linear correlations.

## Results

### Optimising a Method for Quantification of ^18^F-DOPA Uptake in the Midbrain

Based on the time activity curves (TAC; change in SUV across the scan period), the midbrain shows a similar pattern of ^18^F-DOPA uptake compared to the striatum, indicating that the radiotracer behaves in a similar way between these regions (Supp. Figure S2). For instance, the average time at which the TAC peaked (SUV_peak_) was not significantly different between the striatum (100 ± 22.3 min) and midbrain (95 ± 22.3 min; *t*_(22)_ = 0.45, *p* = 0.65; Supp. Figure S2a,b). The slope of the SUV_ratio_ (the cerebellar SUV relative to the SUV of the ROI) was significantly different between the midbrain and striatum (*F* = 240.9, *p* < 0.0001); however, both were linear (striatum *r*^2^ =0.88, midbrain *r*^2^ = 0.61; Supp. Figure S2c).

By comparing different T*–*T*^end^ windows to calculate *K*_i_^Mod^, we found that the lowest CV% values were acquired when the T*–*T*^end^ window omitted the first 5 min of the scan (Table 2). SD values generally decreased with an earlier T* time; however, the lowest SD for midbrain *K*_i_^Mod^ which also permitted the inclusion of 12/12 animals were acquired using T* times from 10 min upwards. Although data up to 120 mins showed the lowest CV%, the modelling was successful for more individuals (12/12 animals) when data was included up to 140 min. As T* increased (with a *T*^end^ of 140 min), CV% decreased while SD increased. The SD of the *k*_loss_ parameter was also lowest when data was included up to 140 min, and again was lowest using T* times from 10 min upwards (Supp. Table S2).

**Table 2:** *K*_i_^Mod^ values for the midbrain using a range of T*–*T*^end^ intervals

	*T*^end^								
	90			120			140		
T*	Mean (SD)	CV%	*n*	Mean (SD)	CV%	*n*	Mean (SD)	CV%	*n*
5	0.005 (0.0047)	43.1	7	0.0041 (0.0041)	31.7	10	0.0042 (0.0041)	25.4	11
10	0.005 (0.0038)	23.3	11	0.0049 (0.0036)	12.3	11	0.0052 (0.0037)	12.9	12
15	0.012 (0.0097)	19.6	11	0.0064 (0.0047)	11.1	11	0.0064 (0.0045)	12.7	12
20	0.012 (0.013)	21.3	11	0.0075 (0.0059)	11.5	11	0.0072 (0.0050)	12.0	12
25	0.021 (0.018)	20.5	11	0.0083 (0.0069)	11.8	11	0.0078 (0.0052)	11.8	12
30	0.026 (0.026)	24.8	11	0.011 (0.017)	16.7	12	0.0085 (0.0062)	11.5	12

*SD* standard deviation, *CV* coefficient of variation, *n* number out of the full cohort (12) for which the modelling was successful

### Using the Test–Retest Cohort to Assess Consistency for Estimating Midbrain K_i_^Mod^

**Fig. 2:**
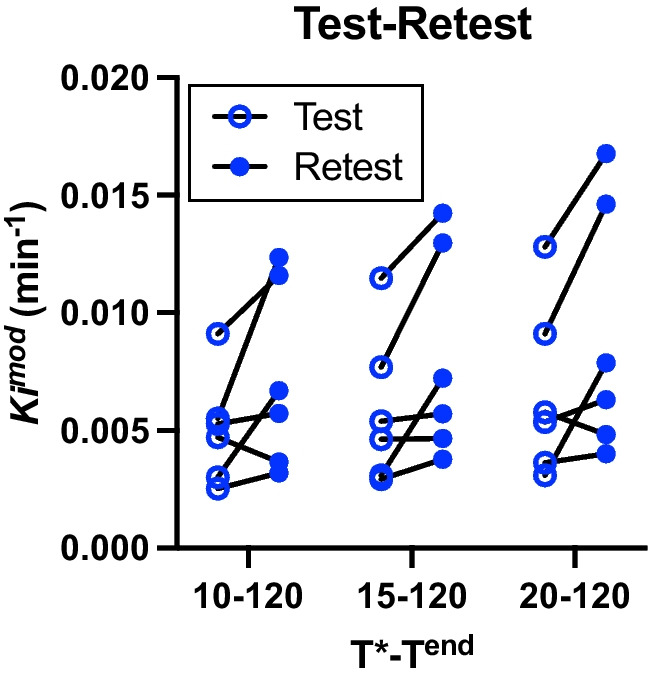
Comparison of *K*_i_^Mod^ values for the test-retest cohort, for a subset of T*–*T*^end^ time windows. *N* = 6

The ICC coefficient values for midbrain *K*_i_^Mod^ from the test–retest cohort were compared for a subset of T*–*T*^end^ windows (Fig. 2). The ICC coefficient for the 10–120 min T*–*T*^end^ window was 0.53 and was 0.74 for both the 15–120 and 20–120 min T*–*T*^end^ windows. However, the absolute variability between test and retest values was lowest for the 15–120 minute T*–*T*^end^ window (43.7% ± 50.1%), compared to the 20–120 minute T*–*T*^end^ window (48.5 ± 55.0%) and the 10–120 min T*–*T*^end^ window (55.2 ± 53.1%).

### Summary of Parameter Testing and Comparison to Analysis in the Striatum

**Fig. 3:**
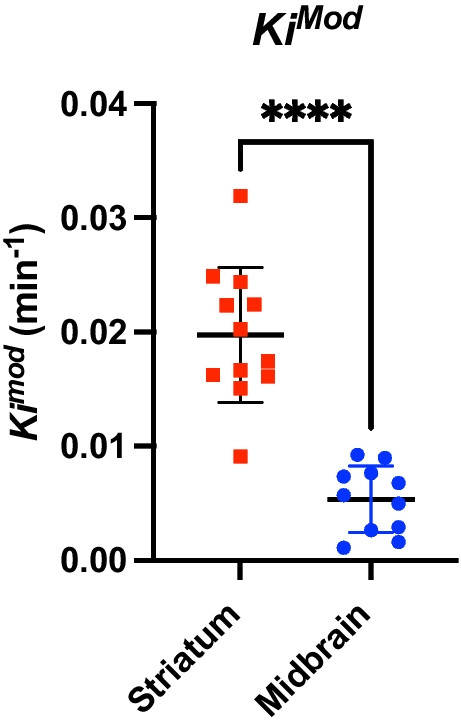
*K*_i_^Mod^ in the striatum and midbrain. *K*_i_^Mod^ was significantly decreased in the midbrain (using the 15–140 min T*–*T*^end^ window) compared to the striatum (using the 20–140 min T*-*T*^end^ window). ****p < 0.0001. Mean ± SD. *n*_(striatum)_ = 12, *n*_(midbrain)_ = 11

Based on the above results, we determined that a T*–*T*^end^ window of 15–140 min was optimal for quantification of ^18^F-DOPA signal in the midbrain. This resulted in one outlier for the analysis of the midbrain, which was removed for analysis. We next compared outputs for the midbrain with the striatum. Previous work has determined that the optimal T*–*T*^end^ window for analysis of *K*_i_^Mod^ in the striatum with ^18^F-DOPA is 20–140 min [8]. *K*_i_^Mod^ in the midbrain (using a 15–140 min T*–*T*^end^ window) showed significantly decreased variability (based on SD) compared to the striatum (using a 20–140 min T*–*T*^end^; *F*_(11)_ = 4.1, *p* = 0.03; Fig. 3). Additionally, the test–retest analysis resulted in a lower ICC coefficient for the striatum (0.56) compared to the midbrain (0.74). However, *K*_i_^Mod^ was significantly lower in the midbrain compared to the striatum (striatum *K*_i_^Mod^ 0.019 ± 0.006, midbrain *K*_i_^Mod^ 0.005 ± 0.003; *t*_(16.3)_ = 7.4, *p* < 0.0001; Fig. 3). There was also a significant decrease in how well the data from the midbrain fitted to the modified Patlak model compared to the striatum (striatum CV% 5.3 ± 1.93%, midbrain CV% 8.4 ± 4.4%; *t*_(21)_ = 2.2, *p* = 0.037; Supp. Figure S3), suggesting that the signal from the midbrain may be noisier than in the striatum. The midbrain also showed significantly decreased *k*_loss_ in comparison to the striatum (*t*_(21)_ = 2.8, *p* = 0.01; Supp. Figure S4).

### Midbrain DA Synthesis Capacity is Increased in the Sub-Chronic Ketamine Model

**Fig. 4:**
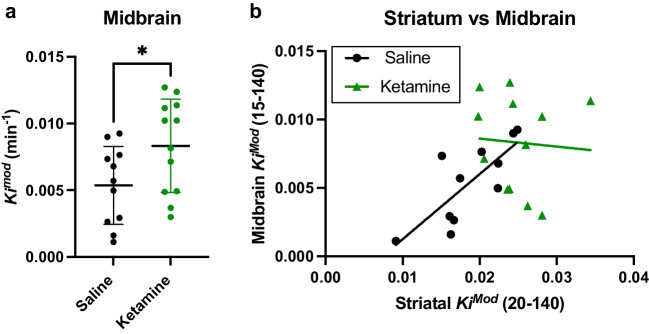
The effect of sub-chronic ketamine treatment on *K*_i_^Mod^ in the midbrain. **a** *K*_i_^Mod^ was elevated in the midbrain of ketamine-treated animals compared to saline-treated animals (*p* = 0.04). **b** There was a significant positive correlation between *K*_i_^Mod^ values for the striatum and midbrain for each animal in the saline-treated group (*p* = 0.005). This was absent in animals which had been treated with ketamine for 5 days (*p* = 0.83). Each data-point represents an individual animal. *p < 0.05. Mean ± SD. *n*_(saline)_ = 11, *n*_(ketamine)_ = 12

We next applied this midbrain method to the sub-chronic ketamine model. There was no significant difference in animal weight, or the ^18^F-DOPA dose injected between the saline- and ketamine-treated groups (Table 1). There was a significant increase in *K*_i_^Mod^ in the striatum of the ketamine-treated animals compared to those treated with saline (*t*_(22)_ = 2.48, *p* = 0.02; Supp. Figure S5). This replicates the previous analysis of this dataset [8]. We found elevated *K*_i_^Mod^ in the midbrain for animals treated with ketamine compared to those treated with saline (*t*_(21)_ = 2.18, *p* = 0.04*;* Fig. 4a). There was also a significant positive correlation between *K*_i_^Mod^ in the striatal and midbrain compartments in saline-treated animals (*r*^2^ = 0.59*, p* = 0.005*;* Fig. 4b). However, this relationship was absent in animals which had been treated with ketamine (*r*^2^ = 0.004, *p* = 0.83). To assess the magnitude of change in the striatum relative to the midbrain, individual *K*_i_^Mod^ values from ketamine-treated animals were compared to the mean values for the control animals in each region. Compared to the mean of the saline-treated group, ketamine-treated animals showed an increased *K*_i_^Mod^ of 26% in the striatum (± 20.1%), and 55% in the midbrain (± 65.3%; Supp. Figure S6). Although these mean values were not statistically different (*t*_(11.6)_ = 1.36, *p* = 0.19, with Welch’s correction), the change in *K*_i_^Mod^ in the midbrain as a result of ketamine treatment was significantly more variable compared to the striatum (*F*_(10)_ = 10.8, *p* = 0.0005).

## Discussion

We found that it is possible to estimate ^18^F-DOPA uptake in the mouse midbrain with moderate test–retest agreement, using an atlas-based template and a T*–*T*^end^ window of 15–140 min. DA synthesis capacity in the mouse midbrain was approximately a quarter of that in the striatum. This is consistent with *ex vivo* work which has shown that levels of the tyrosine hydroxylase (TH) protein are lower in the midbrain compared to the dorsal striatum [18]. TH is the rate-limiting enzyme in the synthesis of DA, and this is therefore a likely explanation for this regional difference in the rate of ^18^F-DOPA uptake we observed. This relative difference in DA synthesis capacity between these regions has also been found in human studies using ^18^F-DOPA PET [13, 27]. The study by Howes and colleagues also found that healthy people demonstrated a positive correlation between nigral and striatal DA synthesis capacity [13], which was evident in the saline-treated mice. While not conclusive, this concordance between human and mouse studies of nigral and striatal ^18^F-DOPA uptake supports the validity of the preclinical method developed here.

Sub-chronic administration of ketamine has been shown to increase DA synthesis capacity in the striatum [6]. Here, we show that this model also results in an elevated DA synthesis capacity in the midbrain. Although we were not able to conduct an *ex vivo* analysis of the cohort which underwent PET scans here, another study in which ketamine (10 mg/kg) was delivered to mice daily for 7 days found increased TH protein levels in the VTA [28]. A separate study found that repeated ketamine exposure increased the firing activity of DA neurons in the VTA [29]. These studies provide indirect support for our finding of elevated DA synthesis capacity in the midbrain following sub-chronic ketamine administration.

This finding in the sub-chronic ketamine model of schizophrenia also appears to replicate the findings in the clinical population; an ^18^F-DOPA PET study found that DA synthesis capacity was elevated in both the nigra and striatum of people with schizophrenia, compared to healthy controls [13]. However, it should be noted that another study found no difference between ^18^F-DOPA in the nigra for patients with schizophrenia and healthy controls, although this study may have been underpowered to detect a difference in this region [19]. Another study which assessed dopaminergic activity in the nigra found blunted amphetamine-induced DA release in this region [30] which would suggest diminished DA synthesis capacity. However, this study used a radiotracer which binds to dopamine 2/3 receptors, and these results therefore cannot be directly compared to those acquired with ^18^F-DOPA.

There is additional indirect evidence that dysfunctional dopaminergic transmission is present in the nigra of people with schizophrenia. Although a recent review of post-mortem studies found no clear difference in levels of midbrain TH in the patient population [15], it is possible to use magnetic resonance imaging to assess levels of neuromelanin—an accumulated product of cytosolic DA and DOPA—in the living brain [31]. A recent meta-analysis of these studies found that neuromelanin levels in the nigra were elevated in people with schizophrenia, compared to healthy controls [14]. The current work therefore supports the theory of hyperdopaminergia in the midbrain as a component of schizophrenia neurobiology.

The exact mechanism by which midbrain DA is elevated in this sub-chronic ketamine model is still unclear. Previous work in the sub-chronic ketamine model has found decreased parvalbumin (PV) interneuron number in the prelimbic cortex and in a subregion of the hippocampus, and that the activation of PV interneurons could ameliorate the behavioural and neurochemical deficits in this model [6]. This reduction in PV interneuron number may contribute to the disinhibition of excitatory glutamatergic input to the midbrain [32]. This in turn may result in elevated firing rates of DA neurons in the midbrain. A decrease in mRNA levels of parvalbumin has also been found in cortical post-mortem tissue from people with schizophrenia [33]. This work is therefore congruent with the hypothesis that striatal hyperdopaminergia in schizophrenia may stem from disinhibition of midbrain DA neurons [34, 35].

Sub-chronic ketamine treatment also disrupted the positive correlation between DA synthesis capacity in the midbrain and nigra which was evident in saline-treated animals. Although there was no significant difference in the magnitude of change induced by ketamine in each region, the change induced by sub-chronic ketamine was more variable in the midbrain compared to the striatum, with some ketamine-treated animals showing a midbrain *K*_i_^Mod^ below the mean of the saline-treated animals. Interestingly, this finding of a disrupted relationship between DA synthesis capacity in these two compartments has also been found in people with schizophrenia [13]. This suggests that the ketamine model can replicate this decoupling between the dopaminergic compartments which is seen in the patient population. This model may therefore be extremely useful for trialling treatments designed to rescue dopaminergic dysfunction throughout the nigro-striatal circuit.

Ketamine is also utilised as an antidepressant [36], and there is some evidence that the positive effects of ketamine are due to increased midbrain DA neuron firing and activity [29, 37]. Our findings provide support for this hypothesis by indicating that sub-chronic ketamine can alter DA synthesis capacity in this region. Future studies could clarify the role of ketamine-induced midbrain hyperdopaminergia on the antidepressant actions of this drug by correlating midbrain DA synthesis capacity with behavioural outputs relevant to depression.

## Limitations

The nigra is situated very close to the midline, and this method therefore uses an ROI which encompassed the bilateral midbrain. As a result, this technique does not allow for studies of lateralization, including the use of unilateral models. The location of the nigra also means that the midbrain ROI may incorporate other monoaminergic regions including the raphe nucleus (serotonergic) and the locus coeruleus (noradrenergic). ^18^F-DOPA signal from these monoaminergic regions may contribute to the findings seen here [27]. Determining the contribution of these regions to this signal may be possible by assessing ^18^F-DOPA signal in the midbrain following a DA-specific lesion. The small size of the nigra also means that partial volume effects may contribute to the *K*_i_^Mod^ values acquired for the midbrain. Additionally, this study uses only male animals. There are known sex-specific effects on the outcomes for ketamine administration [5, 38, 39], and therefore, a comparable study using female animals would be extremely valuable. Finally, performing *ex vivo* confirmation of increased DA synthesis capacity in a cohort of ketamine-treated animals which also underwent ^18^F-DOPA PET scans would directly link the results from this neuroimaging method with a biological phenotype.

## Conclusion

This study developed an atlas-based template to quantify ^18^F-DOPA uptake in the mouse midbrain. This methodological advance will permit the interrogation of the nigro-striatal pathway both in healthy animals and in preclinical models of relevance to schizophrenia and other dopaminergic disorders. We have applied this method to show that the sub-chronic ketamine model results in elevated DA synthesis capacity throughout the nigro-striatal pathway. As a result, we can now utilise the sub-chronic ketamine model to trial novel therapeutics which may act to normalise the midbrain DA function.

### Supplementary Information

ESM 1 (DOCX 5.57 MB)

## Data Availability

The datasets generated and/or analysed in this study, as well as all codes used in the present study, are available from the corresponding author upon reasonable request.

## Abbreviation

AADC: amino acid decarboxylase
COMT: catechol-o-methyltransferase
CV: coefficient of variation
DA: dopamine
DOPA: l-3,4-dihydroxyphenylalanine
DOPAC: 3,4-Dihydroxyphenylacetic acid
^18^F-DOPA: fluorinated l-3,4-dihydroxyphenylalanine
IP: intraperitoneal
Ki: influx constant
MBq: megabecquerel
NMDA: N-methyl-d-aspartate
PET: positron emission tomography
PV: parvalbumin
ROI: region of interest
SD: standard deviation
SNpc: substantia nigra pars compacta
SUV: standardised uptake values
TAAR1: trace amine-associated receptor 1
TAC: time activity curve
TH: tyrosine hydroxylase
VTA: ventral tegmental area
