# Correction: Sub-Chronic Ketamine Administration Increases Dopamine Synthesis Capacity in the Mouse Midbrain: a Preclinical *In Vivo* PET Study

**DOI:** 10.1007/s11307-024-01916-y

**Published:** 2024-04-03

**Authors:** Alice Petty, Anna Garcia-Hidalgo, Els F. Halff, Sridhar Natesan, Dominic J. Withers, Elaine E. Irvine, Michelle Kokkinou, Lisa A. Wells, David R. Bonsall, Sac-Pham Tang, Mattia Veronese, Oliver D. Howes

**Affiliations:** 1 Faculty of Medicine, Imperial College London, Institute of Clinical Sciences, London, UK; 2 Psychiatric Imaging Group, MRC London Institute of Medical Sciences, London, UK; 3 King’s College London, Institute of Psychiatry, Psychology and Neuroscience, London, UK; 4 Metabolic Signalling Group, MRC London Institute of Medical Sciences, London, UK; 5 Invicro, Burlington Danes, Hammersmith Hospital, London, UK; 6 Department of Neuroimaging, Institute of Psychiatry, Psychology and Neuroscience, King’s College London, London, UK; 7 Department of Information Engineering, University of Padua, Padua, Italy; 8 South London and Maudsley NHS Foundation Trust, Camberwell, London, UK; 9 H. Lundbeck A/S, St Albans, AL1 2PS UK

**Correction: Molecular Imaging and Biology (2023) 25:1054-1062**

10.1007/s11307-023-01865-y

The original online version of this article was revised to add the following funding note: This project has received funding from the European Union’s Horizon 2020 research and innovation programme under the Marie Skłodowska-Curie grant agreement No 101028661.

The original article has been corrected.

