# Supplementary material for: Sub-Chronic Ketamine Administration Increases Dopamine Synthesis Capacity in the Mouse Midbrain: a Preclinical In Vivo PET Study
Source: Mol Imaging Biol. 2023 Oct 23;25(6):1054–62. doi: 10.1007/s11307-023-01865-y (PMC10728236; doi:10.1007/s11307-023-01865-y)
Supplement: Supplementary file 1 — (DOCX 5.57 MB) [file 11307_2023_1865_MOESM1_ESM.docx]

**Electronic Supplementary Material**

“Sub-chronic ketamine administration increases dopamine synthesis capacity in the midbrain: a preclinical *in vivo* PET study”

Journal: Molecular Imaging and Biology

Alice Petty^1,2^, Anna Garcia-Hidalgo^1,2^, Els F Halff^2,3^, Sridhar Natesan^2,3^, Dominic J Withers^1,4^, Elaine E Irvine^1,4^, Michelle Kokkinou^1,2^, Lisa A Wells^5^, David R Bonsall^5^, Sac-Pham Tang^5^, Mattia Veronese^6,7^, Oliver D Howes^1,2,3,8,9^

Addresses:

1 Institute of Clinical Sciences, Faculty of Medicine, Imperial College London, London, UK

2 Psychiatric Imaging Group, MRC London Institute of Medical Sciences, London, UK

3 Institute of Psychiatry, Psychology and Neuroscience, King’s College London, London, UK

4 Metabolic Signalling Group, MRC London Institute of Medical Sciences, London, UK

5 Invicro, Burlington Danes, Hammersmith Hospital, London, UK

6 Department of Neuroimaging, Institute of Psychiatry, Psychology and Neuroscience, King’s College London, London, UK

7 Department of Information Engineering, University of Padua, Pauda, Italy

8 South London and Maudsley NHS Foundation Trust, Camberwell, London, UK

9 H. Lundbeck A/S, St Albans AL1 2PS, UK

­­­Supplementary figures:

B)


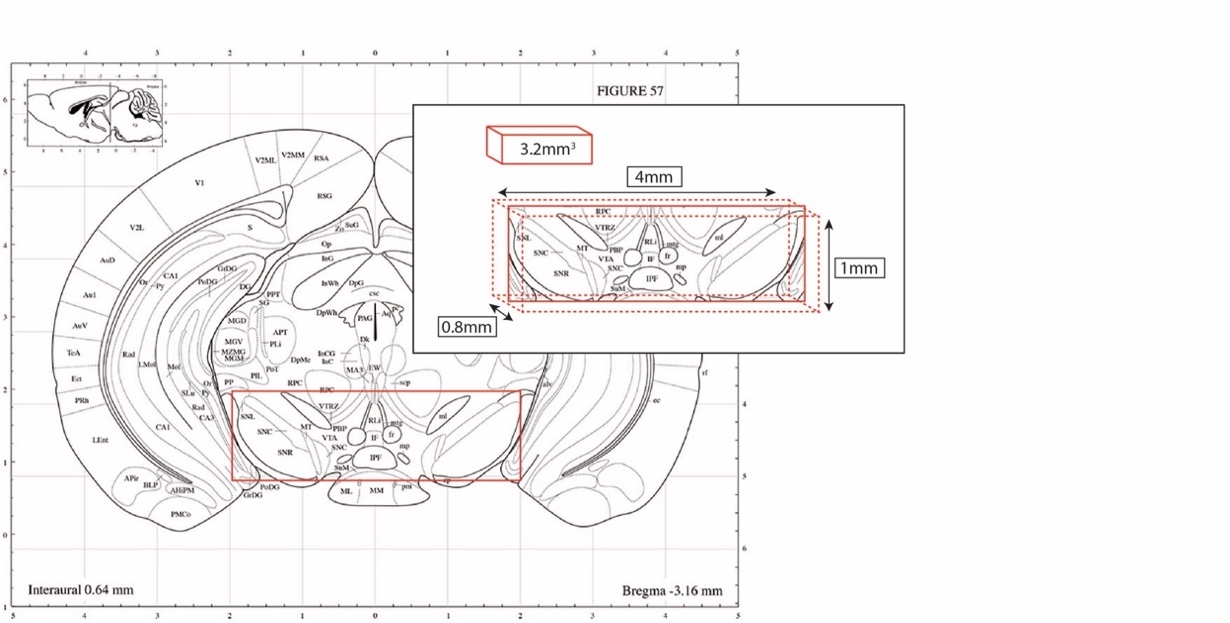


A)


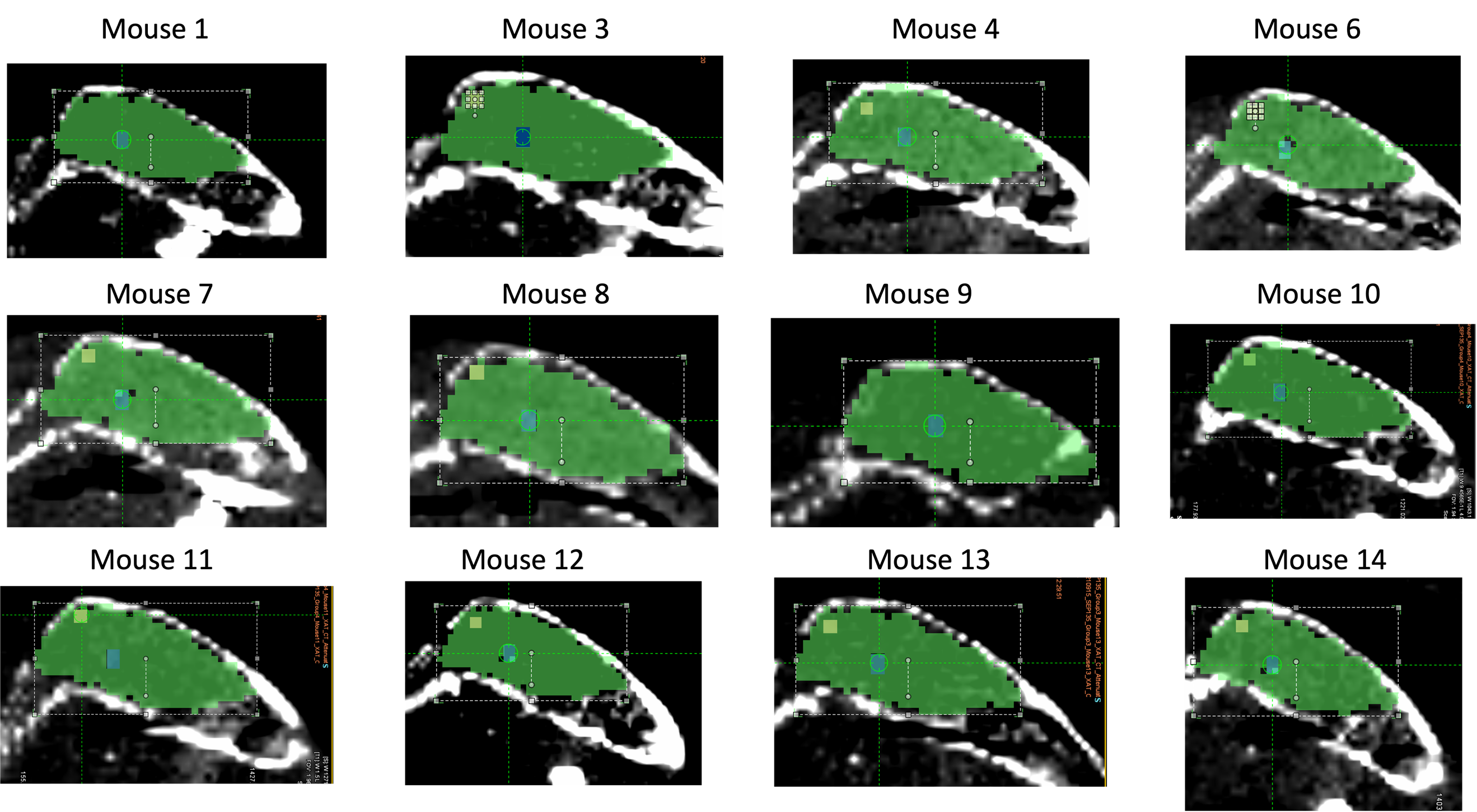


***Figure S1.*** *The location and size of the midbrain ROI. A) The size of the midbrain ROI was determined based on a Mouse Brain Atlas (Keith BJ, Franklin M, Paxinos G (2008) The mouse brain in stereotaxic coordinates, compact: the coronal plates and diagrams. Elsevier science). The position of the midbrain ROI was determined using a template aligned to the sagittal brain view. B) placement of the sagittal template on each of the individual animals from the saline-treated cohort.*

C)

A)

B)

C)

***Figure S2.*** *Comparison of striatal and midbrain time activity curves (TACs). A) The TACs for the midbrain region of interest (ROI) followed a similar pattern to the striatum and cerebellum, and B) the time at which each TAC peaked (SUV_peak_) was not significantly different between the striatum and midbrain (p=0.65). C) The slope of the ratio of the SUV for the striatum and midbrain to the cerebellum (SUV_ratio_) was significantly different between the striatum and midbrain, however all slopes were similar linear (striatum r^2^=0.88, midbrain r^2^=0.61). Mean±SD, n=12.*

**

***Figure S3.*** *Comparison of striatal and midbrain CV% for K_i_^Mod^. Mean CV% was significantly increased in the midbrain (using the 15-140 T*-T^end^ window) compared to the striatum (using a 20-140 T*-T^end^ window; p=0.03), suggesting noisier data from this region. Mean±SD.* n_(striatum)_=12, n_(midbrain)_=11*.*

***Figure S4.*** *K_loss_ in the midbrain and striatum. K_loss_ was significantly decreased in the midbrain compared to the striatum, using the 15-140 minute T*-T^end^ window in the midbrain (p=0.01). Mean± SD.* n_(striatum)_=12, n_(midbrain)_=11.

**

***Figure S5.*** *K_i_^Mod^ in the striatum was increased* *in ketamine-treated animals compared to saline-treated animals (p=0.02). *p<0.05. Mean± SD, n=12 per group.*

******

***Figure S6.*** *The change in K_i_^Mod^ in ketamine-treated animals, relative to the control group mean for each region. The mean magnitude of change was not significantly different between the striatum and midbrain (p=0.16). Mean±SD,* n_(striatum)_=12, n_(midbrain)_=11*.*

Supplementary tables:

**Table S1.** Specifications of the ROI sizes for different regions.

| Region | **X (mm)** | **Y (mm)** | **Z (mm)** | **Volume (mm^3^)** |
| --- | --- | --- | --- | --- |
| *Cerebellum* | 1.33 | 0.89 | 0.89 | 1.1 |
| *Striata* | 0.89 | 0.89 | 0.89 | 0.70 |
| *Midbrain* | 3.11 | 1.33 | 0.89 | 3.7 |

**Table S2.** *k_loss_* values for different T*-Tend windows.

|  | **90** | | **120** | | **140** | |
| --- | --- | --- | --- | --- | --- | --- |
| *T*-T^end^* | *Mean* | *SD* | *Mean* | *SD* | *Mean* | *SD* |
| *5* | 0.021 | 0.037 | 0.0097 | 0.019 | 0.013 | 0.025 |
| *10* | 0.011 | 0.015 | 0.013 | 0.022 | 0.0098 | 0.0093 |
| *15* | 0.040 | 0.041 | 0.019 | 0.028 | 0.013 | 0.011 |
| *20* | 0.030 | 0.038 | 0.021 | 0.029 | 0.014 | 0.013 |
| *25* | 0.051 | 0.044 | 0.023 | 0.030 | 0.015 | 0.014 |
| *30* | 0.047 | 0.048 | 0.018 | 0.027 | 0.016 | 0.015 |
